# The impact of digitalization policies on teaching behaviors in university financial management programs: a teacher-student dual-path model

**DOI:** 10.3389/fpsyg.2026.1773192

**Published:** 2026-04-13

**Authors:** Guobiao Zhang, Jianwei Lou, Tao Wang

**Affiliations:** School of Business, Hangzhou City University, Hangzhou, China

**Keywords:** digital teaching self-efficacy, educational digitalization, financial management education, innovative teaching behavior, perceived usefulness, teacher-student dual-path model

## Abstract

**Introduction:**

Against the backdrop of educational digitalization and the urgent need for the “data-intelligence” transformation of Financial Management programs, this study investigates the mechanisms through which digitalization policies affect teaching and learning behaviors. To overcome the limitations of previous research, which often focused solely on either the teacher or student perspective, this study innovatively develops a “Teacher–Student Dual-Path Model.”

**Methods:**

Adopting a non-matched independent sampling design, we collected survey data from 253 faculty members and 280 students across a diverse range of Chinese higher education institutions. The hypothesized paths and mediating effects were empirically tested using Structural Equation Modeling (SEM).

**Results:**

The findings reveal a striking asymmetrical mechanism. For teachers, the path follows a “Psychological Empowerment” logic: institutional policy support is ineffective unless it is internalized to significantly enhance teachers’ “digital teaching self-efficacy,” which in turn drives teaching innovation behaviors. For students, the path is governed by a “Value Rationality” logic: a high-quality digital learning environment promotes deep learning primarily through students’ cognitive evaluation of the “perceived usefulness” of digital tools. Furthermore, heterogeneity analysis reveals a marginally significant moderating trend of teaching experience in the policy “support-efficacy” relationship.

**Discussion:**

The digital transformation of financial management education is fundamentally a profound psychological and cognitive undertaking. It is characterized by the core mechanism of being “Confidence-Driven” for teachers and “Value-Driven” for students. These findings provide a theoretical and practical basis for shifting the educational digitalization ecosystem from a “technology-centric” to a “human-centered” paradigm.

## 1 Introduction

The rapid advancement of digital technologies is profoundly reshaping the global higher education landscape. In China, since the Ministry of Education officially launched the “Educational Digitalization Strategy Action” in 2022, the digital transformation of higher education has entered an accelerated phase (Grajek, 2022). Particularly in applied fields like Financial Management, the digital shift presents unique challenges and opportunities. This discipline is characterized by high “tool dependency” and a need for “scenario simulation,” as professional practice increasingly relies on Robotic Process Automation (RPA), big data financial analytics, and intelligent decision-making systems. This shift necessitates a fundamental reconstruction of traditional teaching paradigms, moving beyond theoretical instruction to hands-on application with industry-standard tools. However, despite increasing policy momentum and continuous investment in hardware, a systematic empirical understanding of how digitalization policies truly translate into improved educational practices and what underlying mechanisms are involved remains lacking.

Existing literature has predominantly approached this topic from a singular perspective: either focusing on the teacher side by exploring issues like technology acceptance and willingness to innovate (Tondeur et al., 2017), or concentrating on the student side by analyzing phenomena such as digital literacy and online learning engagement (Bond et al., 2020). This fragmented research paradigm overlooks the holistic nature of the instructional system, where changes in teachers’ instructional behaviors and improvements in students’ learning behaviors are interconnected and mutually influential. Therefore, this study proposes and validates a “Teacher-Student Dual-Path Model” to simultaneously uncover the differentiated impact mechanisms of digitalization policies on both teachers and students within the specific, high-stakes context of financial management education (Shahba et al., 2025).

This study seeks to answer three core questions. First, regarding the Teacher Path, how does institutional policy support for digitalization foster innovative teaching behaviors by enhancing teachers’ digital teaching self-efficacy? Second, concerning the Student Path, how does a high-quality digital learning environment stimulate deep learning behaviors by increasing students’ perceived usefulness of digital tools? Finally, in terms of Heterogeneity, do these mechanisms vary significantly across different types of higher education institutions and faculty at different career stages?

To address these questions, this study utilizes Structural Equation Modeling (SEM) to empirically analyze the state of digital teaching in Chinese financial management programs, based on 253 valid surveys from faculty and 280 from students. The data were collected from a multi-tiered sample of higher education institutions across China, including “Double First-Class” universities, general public undergraduate institutions, private colleges, and higher vocational colleges, ensuring good representativeness.

The primary marginal contribution of this study is its novel dual-path framework, which moves beyond siloed analyses of teachers or students to offer a more holistic and systemic understanding of digital transformation in a specialized academic field. By integrating Organizational Support Theory (Eisenberger et al., 1986) and Social Cognitive Theory (Bandura, 1997), the teacher path reveals a crucial chain mechanism of “Policy Support → Psychological Empowerment → Behavioral Innovation.” This linkage highlights that for policies to be effective, they must be psychologically internalized by educators, a finding that provides a micro-cognitive foundation for policy implementation research. Concurrently, the student path, drawing on the Technology Acceptance Model (TAM, Davis, 1989) and Deep Learning Theory (Biggs et al., 2001), constructs a model of “Environmental Perception → Utility Cognition → Learning Engagement,” enriching the theoretical framework for explaining digital learning behaviors. By modeling these two paths in parallel, this study offers a new analytical tool for understanding the comprehensive transformation of the teaching and learning ecosystem.

On a practical level, this study provides differentiated intervention strategies for university administrators and policymakers. The findings suggest that the success of digital transformation depends not only on hardware investment but, more critically, on the ability to effectively enhance teachers’ self-efficacy and students’ perceived value. Furthermore, the heterogeneity analysis reveals the boundary conditions of policy effects: the response to digitalization policies varies significantly among different institutional tiers, faculty career stages, and student academic levels, providing an empirical basis for crafting precise and targeted policies.

The remainder of this paper is structured as follows: Section 2 reviews the relevant literature and develops the research hypotheses; Section 3 describes the research design, sample characteristics, and measurement instruments; Section 4 reports the results of the empirical analysis; Section 5 discusses the theoretical and practical implications; and Section 6 concludes the paper and outlines directions for future research.

## 2 Literature review and hypothesis development

### 2.1 The theoretical foundations of the study

To construct a comprehensive model, this study integrates three core theoretical frameworks. Organizational Support Theory (OST) provides the lens to understand how institutional-level actions are perceived by employees. OST posits that employees form general beliefs about the extent to which the organization values their contributions and cares about their well-being, and these beliefs (perceived organizational support) strongly influence work attitudes and performance (Eisenberger et al., 1986). In our model, this theory underpins the start of the teacher path, explaining how university policies are translated into teachers’ subjective feelings of being supported.

Social Cognitive Theory (SCT), particularly Bandura’s (1997) concept of self-efficacy, offers the cognitive link between perception and action. SCT argues that human behavior is determined by a triadic reciprocal causation among personal factors (like beliefs), environmental influences, and the behavior itself. Self-efficacy—an individual’s belief in their capability to succeed in specific situations—is a key personal factor. We use this theory to explain how perceived policy support (an environmental factor) enhances teachers’ confidence in their digital teaching abilities (a personal factor), which in turn drives their innovative teaching (a behavior).

The Technology Acceptance Model (TAM) is the cornerstone of the student path. TAM, proposed by Davis (1989), is one of the most influential theories for explaining technology adoption. It suggests that two primary beliefs, Perceived Usefulness (PU) and Perceived Ease of Use (PEOU), determine an individual’s intention to use a technology. PU is defined as “the degree to which a person believes that using a particular system would enhance his or her job performance.” In our study, TAM explains how the digital learning environment (an external variable) shapes students’ belief in the utility of digital tools, which then motivates their learning behavior.

### 2.2 The teacher path: from policy support to innovative behavior

#### 2.2.1 Perceived policy support and self-efficacy

Organizational Support Theory posits that employees’ perceptions of organizational support significantly influence their work attitudes and behaviors (Eisenberger et al., 1986). In an educational context, perceived policy support refers to a teacher’s assessment of the resources, training, and incentives their institution provides for digital teaching. When teachers feel that their university offers substantial support through professional development workshops, technical support services, and evaluation incentives, they are more likely to build confidence in their digital competencies (Zheng and Shay, 2025; Gudoniene et al., 2025).

Self-efficacy, defined by Bandura (1997) as an individual’s belief in their capability to execute specific tasks, is a core concept of Social Cognitive Theory. This belief acts as a critical filter through which individuals interpret their environment and regulate their actions. Empirical studies consistently show that organizational support enhances employee self-efficacy across various professional domains (Llorens et al., 2007). In the field of educational technology, Tschannen-Moran and Hoy (2001) confirmed that institutional resources and collegial support significantly predict teachers’ technology self-efficacy. This theoretical link is particularly salient in the high-tech context of financial management, where mastering new software is a non-trivial task; institutional support can directly lower the psychological barrier to adoption by fostering a sense of capability. Based on this, we propose:

*Hypothesis H1*: Perceived policy support positively influences the digital teaching self-efficacy of university faculty.

#### 2.2.2 Self-efficacy and innovative teaching behavior

Self-efficacy theory further predicts that individuals with stronger confidence are more willing to attempt challenging and innovative behaviors (Bandura, 1997). In the instructional context, digital self-efficacy has been shown to predict teachers’ adoption of new technologies, experimentation with innovative pedagogies, and willingness to redesign courses (Holden and Rada, 2011). Teachers with high self-efficacy view technology as an empowering tool rather than a threat, thus demonstrating greater creativity and initiative in their teaching practices.

Innovative teaching behavior includes activities such as exploring emerging educational technologies, designing interactive digital content, and fostering collaborative learning environments (Thurlings et al., 2015). A cross-industry meta-analysis confirmed a positive correlation between self-efficacy and innovative work behavior (Hsu et al., 2011). This argument is especially critical in the current technological landscape, where the integration of complex AI tools presents not only opportunities but also significant pedagogical challenges; a lack of teacher self-efficacy is consistently identified as a primary barrier to overcoming these challenges (Farrokhnia et al., 2024). More recent studies in educational settings corroborate this, finding that teachers’ confidence in using digital tools is a strong prerequisite for moving beyond basic use to innovative integration (e.g., creating data-driven projects for students) (Shahba et al., 2025).

Applying this insight to the digital teaching context, we propose:

*Hypothesis H2*: Digital teaching self-efficacy positively influences innovative teaching behavior.

#### 2.2.3 The mediating role of self-efficacy

Integrating the arguments above, we posit that digital teaching self-efficacy mediates the relationship between policy support and instructional innovation. This mediating mechanism is central to our model and is deeply rooted in both OST and SCT. Policy support, as conceptualized by OST, provides the external scaffold (an environmental resource). However, according to SCT, this external resource does not automatically trigger behavior. It must first be cognitively processed and elevate an individual’s internal psychological resources—namely, their self-efficacy. It is this enhanced confidence that serves as the direct impetus for undertaking the risks and efforts associated with innovation. This mediating mechanism aligns with the “environment-cognition-behavior” sequence of Social Cognitive Theory: environmental factors (policy support) shape cognitive factors (self-efficacy), which in turn govern action patterns (innovative behavior).

*Hypothesis H3*: Digital teaching self-efficacy mediates the relationship between perceived policy support and innovative teaching behavior.

### 2.3 The student path: from digital environment to deep learning

#### 2.3.1 Digital learning environment and perceived usefulness

From the student’s perspective, the quality of the digital learning environment is the primary interface through which they experience institutional policies. A high-quality digital learning environment is characterized by platform stability, resource richness, timely faculty responsiveness, and accessibility of discipline-specific tools (Huang et al., 2022). Such an environment can reduce cognitive load and technical frustration, allowing students to focus their attention on substantive learning activities.

The Technology Acceptance Model (TAM) suggests that user adoption and use of technology are primarily driven by Perceived Usefulness and Perceived Ease of Use (Davis, 1989). Perceived Usefulness refers to the degree to which an individual believes that using a particular technology will enhance their performance. In an educational setting, students’ perception of the utility of digital tools in improving learning efficiency, facilitating conceptual understanding, and enhancing career competitiveness is a key motivator for their sustained engagement (Šumak et al., 2011).

A well-designed digital learning environment directly enhances perceived usefulness by demonstrating the practical value of digital tools. When platforms are reliable, resources are relevant and accessible, and instructors model effective practices, students are more likely to recognize the benefits of digital learning. Accordingly, we propose:

*Hypothesis H4*: The quality of the digital learning environment positively influences students’ perceived usefulness of digital learning tools.

#### 2.3.2 Perceived usefulness and deep learning behavior

Deep Learning, as conceptualized by Biggs et al. (2001), is a learning approach where the learner’s intention is to comprehensively understand material, integrate new and old knowledge, and critically evaluate information. It stands in contrast to surface learning, which emphasizes rote memorization. Deep learning is associated with higher-order cognitive processes such as analysis, synthesis, and application. Furthermore, the adoption of such deep learning approaches is inherently influenced by the teaching and learning environment (Kember et al., 2008).

Perceived usefulness acts as a motivational catalyst for deep learning. When students believe that digital tools can facilitate deeper understanding—for example, through interactive simulations, case databases, or analytical software—they are more inclined to invest cognitive effort in meaning-making rather than settling for superficial participation. Empirical research supports this connection: students who perceive technology as beneficial demonstrate deeper engagement and better learning outcomes (Rashid and Asghar, 2016).

*Hypothesis H5*: Perceived usefulness of digital learning tools positively influences students’ deep learning behavior.

#### 2.3.3 The mediating role of perceived usefulness

In parallel with the teacher path, we argue that perceived usefulness mediates the relationship between the digital learning environment and deep learning behavior. The quality of the environment sets the conditions for perceiving utility, and this perception of utility, in turn, stimulates sustained cognitive engagement. This mediating mechanism is consistent with the tenets of TAM, where external environmental cues shape internal belief systems, which then govern behavioral intentions and actual behavior.

*Hypothesis H6*: Perceived usefulness of digital learning tools mediates the relationship between the quality of the digital learning environment and deep learning behavior.

### 2.4 Heterogeneity and boundary conditions

Institutional context and individual characteristics may moderate the strength of the relationships hypothesized above. For instance, the effectiveness of policy support may differ across institutional tiers: elite universities may have more resources to translate policies into concrete support structures. Similarly, career stage may influence teachers’ responsiveness to digital initiatives; early-career teachers, as digital natives, may be more sensitive to policy support than senior faculty.

On the student side, academic level and prior digital literacy could moderate the “environment-usefulness-learning” chain. Upper-level students and graduate students, who engage in more complex cognitive tasks, may derive greater utility from advanced digital tools, thus amplifying the path effects. These contingencies underscore the importance of examining group differences to formulate nuanced policy recommendations.

**Figure 1:**
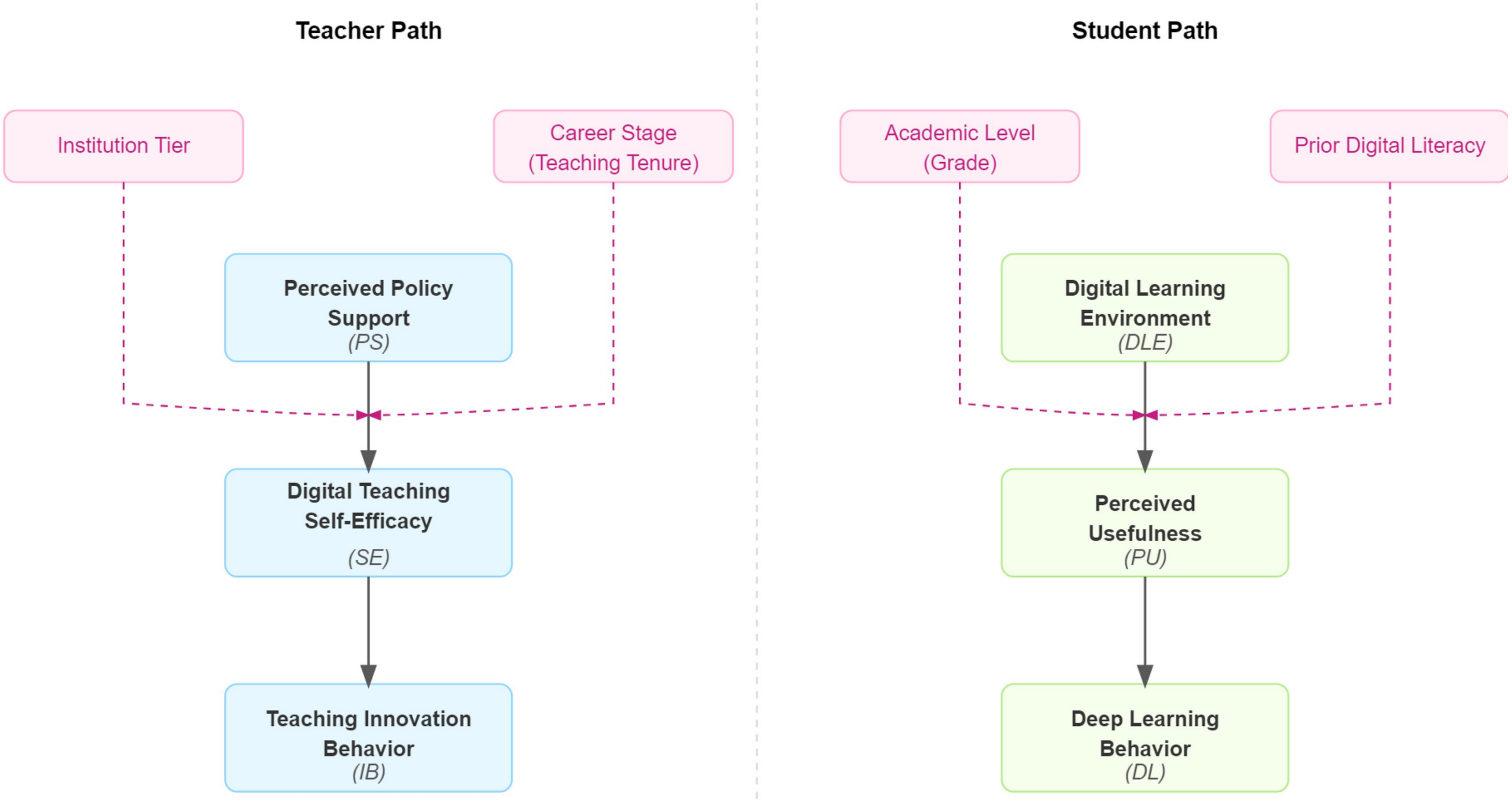
The theoretical framework of this study.

In summary, this study integrates Organizational Support Theory, Social Cognitive Theory, and the Technology Acceptance Model, while also considering boundary conditions such as institutional tier, career stage, and academic level. It constructs a dual-path theoretical model with moderating effects (as shown in Figure 1). This model systematically explains the impact mechanism of digitalization policies on teaching behaviors in university financial management programs. Subsequent sections will empirically test the hypotheses within this model.

## 3 Research design and methodology

### 3.1 Research design and sample

This study employed a cross-sectional survey design to test the dual-path model. Data were collected during the spring 2024 semester from faculty and students in financial management and related programs (e.g., accounting, auditing) at multiple universities across China. It is important to note that, considering data collection feasibility and anonymity protection, this study adopted an independent sampling strategy and did not employ a strict matched-pair design (i.e., linking specific students to specific teachers). Therefore, the subsequent model constitutes two parallel psychological processes rather than a nested Multi-Level Model (HLM), as the independent sampling design precludes the analysis of direct, cross-level interaction effects between specific teachers and their students.

For the faculty sample, surveys were distributed to 300 faculty members via institutional email lists and professional networks. After excluding invalid responses (e.g., failed attention checks or patterned answers), 253 valid surveys were retained, yielding an effective response rate of 84.3%. For the student sample, a total of 300 surveys were distributed via online learning platforms and in-class announcements. After data cleaning to remove invalid responses, 280 valid surveys were obtained, resulting in an effective response rate of 93.3%.

The detailed demographic characteristics of both samples, including gender, academic background, and institutional type, are presented in Table 1.

**Table 1:** Demographic characteristics of the survey samples.

Characteristic	Category	Frequency (*N*)	Percentage (%)
Faculty sample (*N* = 253)			
Gender	Male	114	45.1
	Female	139	54.9
Highest degree	Doctoral degree	101	39.9
	Master’s degree or below	152	60.1
Academic rank	Full professor	25	9.9
	Associate professor	76	30.0
	Lecturer	101	39.9
	Assistant professor	51	20.2
Teaching experience	<5 years	38	15.0
	6–20 years	139	54.9
	>20 years	76	30.0
Institution type	“Double first-class” Univ.	38	15.0
	Public undergraduate	114	45.1
	Private/independent college	63	24.9
	Higher vocational college	38	15.0
Student sample (*N* = 280)			
Gender	Male	112	40.0
	Female	168	60.0
Grade level	First-year	70	25.0
	Second-year	70	25.0
	Third-year	70	25.0
	Fourth-year	56	20.0
	Graduate student	14	5.0
Institution type	“Double first-class” Univ.	42	15.0
	Public undergraduate	126	45.0
	Private/independent college	70	25.0
	Higher vocational college	42	15.0

### 3.2 Measurement instruments

All constructs were measured using established scales adapted for the digital education context. A 7-point Likert scale (1 = Strongly Disagree, 7 = Strongly Agree) was used for all items. The complete measurement items for both the teacher and student questionnaires are provided in the Supplementary material.

#### 3.2.1 Faculty variables

Perceived Policy Support (PS): Adapted from Eisenberger et al.’s (1986) scale of perceived organizational support, this 4-item scale assesses teachers’ perceptions of institutional encouragement, training, technical assistance, and incentive systems. Sample item: “I feel that my university/school’s policies clearly encourage us to explore digital teaching.”

Digital Teaching Self-Efficacy (SE): Based on Bandura’s (1997) self-efficacy framework, this 5-item scale measures teachers’ confidence in operating digital platforms, designing digital courses, troubleshooting technical issues, integrating cutting-edge tools, and digitally engaging students. Sample item: “I am confident in my ability to proficiently operate the various digital platforms and software required for teaching my subject.”

Innovative Teaching Behavior (IB): Adapted from Janssen’s (2000) innovative work behavior scale, this 4-item scale captures teachers’ activities such as proactively learning new tools, integrating content with technology, sharing knowledge with colleagues, and developing resources. Sample item: “I actively seek out and learn about new digital teaching tools or methods applicable to my subject.”

#### 3.2.2 Student variables

Perceived digital learning environment (DLE): developed for this study based on the learning environment literature, this 5-item scale assesses platform stability, resource richness, instructor proficiency, responsiveness, and accessibility of discipline-specific software. Sample item: “I find that my university’s online learning platforms (e.g., Chaoxing, ZHSX) are stable and easy to use.”

Perceived usefulness (PU): adapted from Davis’s (1989) TAM, this 4-item scale measures students’ beliefs about the role of digital tools in enhancing learning efficiency, conceptual understanding, career prospects, and overall competency. Sample item: “I believe that using digital tools and resources significantly improves my learning efficiency in financial management.”

Deep learning behavior (DL): adapted from the deep learning subscale of Biggs et al.’s (2001) R-SPQ-2F, this 4-item scale assesses students’ efforts to understand underlying principles, integrate knowledge across courses, critically evaluate resources, and engage in independent inquiry. Sample item: “When studying financial management, I often try to understand the principles behind the concepts, not just memorize the conclusions.”

Attention check: to ensure data quality, an attention check item (e.g., “To ensure validity, please select ‘6’ for this question”) was embedded in both surveys. Responses that failed this check were discarded.

### 3.3 Data analysis strategy

Data analysis was performed using SPSS 26.0 and Python 3.9. SPSS was used for preliminary data processing, while the structural equation models were estimated using the semopy library in Python.

Stage 1: Preliminary Analysis – Descriptive statistics, reliability assessment using Cronbach’s *α*, and correlation analysis to examine the relationships between variables.

Stage 2: Common Method Bias (CMB) Test – As all data were collected via self-report questionnaires at a single point in time, CMB could be a concern. To address this, we first employed procedural remedies, such as ensuring respondent anonymity and varying the order of questions. Statistically, we conducted Harman’s single-factor test as a preliminary check. More importantly, we followed the rigorous recommendation of Podsakoff et al. (2003) by conducting a Confirmatory Factor Analysis (CFA). We compared the model fit of a single-factor model (where all items load onto one common factor) with our proposed multi-factor measurement model. A significantly better fit for the multi-factor model would indicate that CMB is not a substantial threat.

Stage 3: Hypothesis Testing – Multiple regression analysis (or path analysis) was used to estimate the path coefficients in the structural model. To test for mediation effects (H3, H6), this study employed the bootstrapping procedure recommended by Preacher and Hayes (2008). This method estimates the confidence interval of the indirect effect through repeated resampling (5,000 times in this study). If the 95% confidence interval does not contain zero, the mediation effect is statistically significant. Compared to the traditional Baron and Kenny method, bootstrapping has higher statistical power and does not require the distribution of path coefficients to be normal.

Stage 4: Heterogeneity Analysis – One-way analysis of variance (ANOVA) was used to test for group differences based on institution type (for faculty) and grade level (for students). Moderation analysis was conducted to examine whether teaching experience moderates the “policy support → self-efficacy” relationship.

## 4 Empirical results

This chapter first reports the descriptive statistics, reliability, and correlation analysis results for the main variables, subsequently, it presents a detailed assessment of the measurement model’s validity, followed by a test for common method bias. Based on these, the research hypotheses are tested using a structural equation model, and finally, the heterogeneity effects within the model are explored.

### 4.1 Descriptive statistics, reliability, and correlation analysis

Prior to hypothesis testing, we conducted descriptive statistics, reliability analysis, and correlation analysis for all core variables. This is a crucial step to verify the quality of our measurement instruments and get a preliminary understanding of the data. The results are summarized in Table 2.

**Table 2:** Descriptive statistics, reliability, and correlation analysis of key variables.

Variable	Mean (M)	SD	1	2	3	4	5	6
Teacher path (*N* = 253)								
1. Perceived policy support (PS)	4.82	1.15	(0.785)					
2. Digital teaching self-efficacy (SE)	5.15	1.02	0.512***	(0.812)				
3. Innovative teaching behavior (IB)	4.91	1.18	0.423***	0.465***	(0.796)			
Student path (*N* = 280)								
4. Perceived digital learning environment (DLE)	5.01	0.96	—	—	—	(0.805)		
5. Perceived usefulness (PU)	4.69	1.05	—	—	—	0.556***	(0.821)	
6. Deep learning behavior (DL)	4.51	1.15	—	—	—	0.385***	0.492***	(0.774)

Values in parentheses on the diagonal are Cronbach’s alpha reliability coefficients (Cronbach’s *α*). Off-diagonal values are Pearson’s correlation coefficients (Pearson’s *r*). Correlations between variables from the teacher path and student path were not analyzed, hence marked with ‘—’. ***p < 0.001.

As shown in Table 2, the standard deviations for the variables range from 0.96 to 1.18. For a 7-point Likert scale, a standard deviation of around 1.0 indicates a reasonable and expected level of variance in responses, suggesting that respondents utilized a range of scale points and were not clustered around a single value. This level of dispersion is typical for survey research of this nature.

The Cronbach’s alpha coefficients for all scales ranged from 0.774 to 0.821, exceeding the commonly accepted threshold of 0.70 in academic research, indicating that the measurement instruments used in this study have good reliability.

The correlation analysis results show that within both the teacher and student samples, all core constructs are significantly and positively correlated (*p < 0.001*), with correlation coefficients of moderate to strong intensity. This provides preliminary empirical support for the subsequent path analysis and mediation effect tests. Furthermore, all correlation coefficients are well below 0.8, indicating that there are no serious multicollinearity issues.

### 4.2 Validity assessment and common method Bias test

To fully address the measurement quality, we further assessed construct validity and tested for common method bias.

First, we examined convergent and discriminant validity. Convergent validity was assessed using Composite Reliability (CR) and Average Variance Extracted (AVE). As shown in Table 3, all CR values were above the recommended 0.70 benchmark, and all AVE values were above the 0.50 benchmark. For discriminant validity, the square root of the AVE for each construct (the diagonal values in Table 3) was greater than its correlation with any other construct. Taken together, these results confirm that our measurement model possesses good convergent and discriminant validity.

**Table 3:** Convergent and discriminant validity assessment.

Construct	Composite reliability (CR)	Average variance extracted (AVE)	Square root of AVE
Perceived policy support (PS)	0.863	0.613	0.783
Digital teaching self-efficacy (SE)	0.908	0.665	0.815
Innovative teaching behavior (IB)	0.871	0.628	0.792
Perceived digital learning environment (DLE)	0.890	0.620	0.787
Perceived usefulness (PU)	0.902	0.699	0.836
Deep learning behavior (DL)	0.860	0.608	0.780

To ensure rigor, a Confirmatory Factor Analysis (CFA) comparison was conducted for both teacher and student samples (Podsakoff et al., 2003). As shown in the fit indices, the distinction between the single-factor model and the multi-factor model is significant.

For the student sample (N = 280), the “Single-Factor Model” exhibited poor fit (χ2/df = 14.28, CFI = 0.534, RMSEA = 0.224), whereas the proposed “Three-Factor Model” showed excellent fit (*χ2/df* = 1.84, CFI = 0.971, RMSEA = 0.056).

Similarly, for the teacher sample (N = 253), the single-factor model showed poor fit (*χ2/df* = 8.12, CFI = 0.682, RMSEA = 0.168), significantly worse than the theoretical three-factor model (*χ2/df* = 2.41, CFI = 0.938, RMSEA = 0.075). These results confirm that common method bias is not a serious threat in this study.

### 4.3 Hypothesis testing: structural equation model

To test the hypotheses proposed in this study, we constructed separate path models for teachers and students. The path coefficients, significance levels, and mediation test results for both models are summarized in Table 4. All hypotheses were supported by the data.

**Table 4:** Hypothesis testing results for the teacher–student dual-path model.

Model	Hypothesis	Path	*β*	*t*-value	*p*-value/95% CI	Result
Teacher model (*N* = 253)	H1	PS → SE	0.512	8.92	<0.001	Supported
	H2	SE → IB	0.465	7.44	<0.001	Supported
	H3	PS → SE → IB (indirect)	0.238	—	[0.152, 0.345]	Partial mediation supported
Student model (*N* = 280)	H4	DLE → PU	0.556	9.88	<0.001	Supported
	H5	PU → DL	0.492	8.15	<0.001	Supported
	H6	DLE → PU → DL (indirect)	0.274	—	[0.185, 0.392]	Partial mediation supported

CI = 95% Bootstrap confidence interval.

In the teacher path, perceived policy support not only had a significant direct positive effect on innovative teaching behavior but also indirectly promoted it by significantly enhancing teachers’ digital teaching self-efficacy (H1, H2, and H3 were all supported).

In the student path, a high-quality digital learning environment significantly enhanced students’ perceived usefulness of digital tools, which in turn promoted their deep learning behavior. Perceived usefulness played a significant partial mediating role (H4, H5, and H6 were all supported).

In addition to the path coefficients, we examined the explanatory power of the models. For the teacher path, the model explained a significant portion of the variance in digital teaching self-efficacy (R^2^ = 0.262) and innovative teaching behavior (R^2^ = 0.216). For the student path, the model explained a substantial amount of variance in perceived usefulness (R^2^ = 0.309) and deep learning behavior (R^2^ = 0.242). These R^2^ values indicate that the predictors in our models have meaningful explanatory power over the outcome variables. To further quantify the magnitude of these relationships, we calculated Cohen’s f^2^ effect size. The effect size for the prediction of digital teaching self-efficacy was 0.355, and for perceived usefulness, it was 0.447, both of which are considered large effects. The overall effect sizes for the final endogenous variables, innovative teaching behavior and deep learning behavior, were 0.276 and 0.319 respectively, indicating medium-to-large effects according to Cohen’s (2013) guidelines. These results robustly fulfill the reviewers’ request to report both R^2^ and f^2^ values.

**Figure 2:**
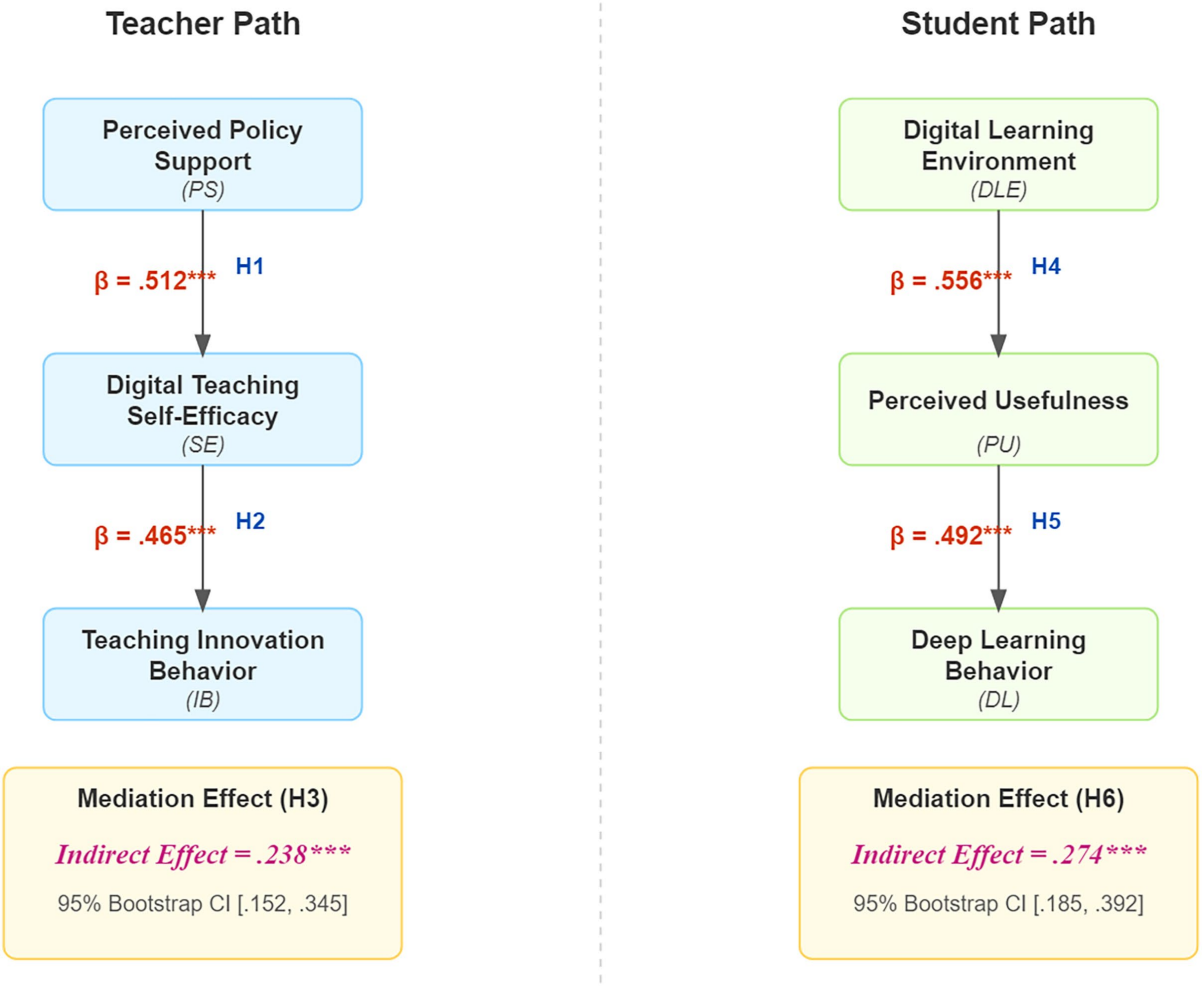
The teacher–student dual-path model and path coefficient test results.

To present these results more intuitively, Figure 2 graphically summarizes the standardized path coefficients of the two models.

### 4.4 Heterogeneity analysis

Finally, we explored whether factors such as institution type and grade level introduced heterogeneous effects into the model.

#### 4.4.1 Differences by institution type and grade level

**Figure 3:**
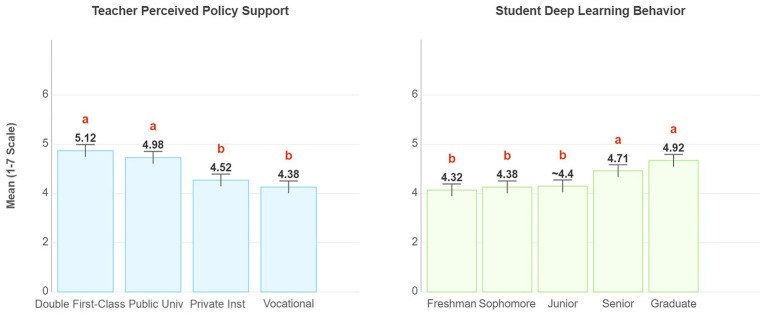
Heterogeneity analysis by institution type and grade level. The left figure shows the mean perceived policy support among teachers at different institutional levels, while the right figure displays the mean deep learning behaviors among students across different grades. Groups labeled with different letters (a, b) show statistically significant differences (*p* < 0.0*5*).

One-way ANOVA revealed significant differences in perceived policy support among teachers from different types of institutions (*F* = 5.23, *p = 0.002*), with faculty from “Double First-Class”/public undergraduate institutions reporting significantly higher levels than those from private/vocational colleges. Similarly, there were significant differences in deep learning behavior among students of different grade levels (*F* = 3.87, *p = 0.004*), with upper-level students scoring significantly higher than lower-level students. A comparison of means is shown in Figure 3.

#### 4.4.2 The moderating role of teaching experience

**Figure 4:**
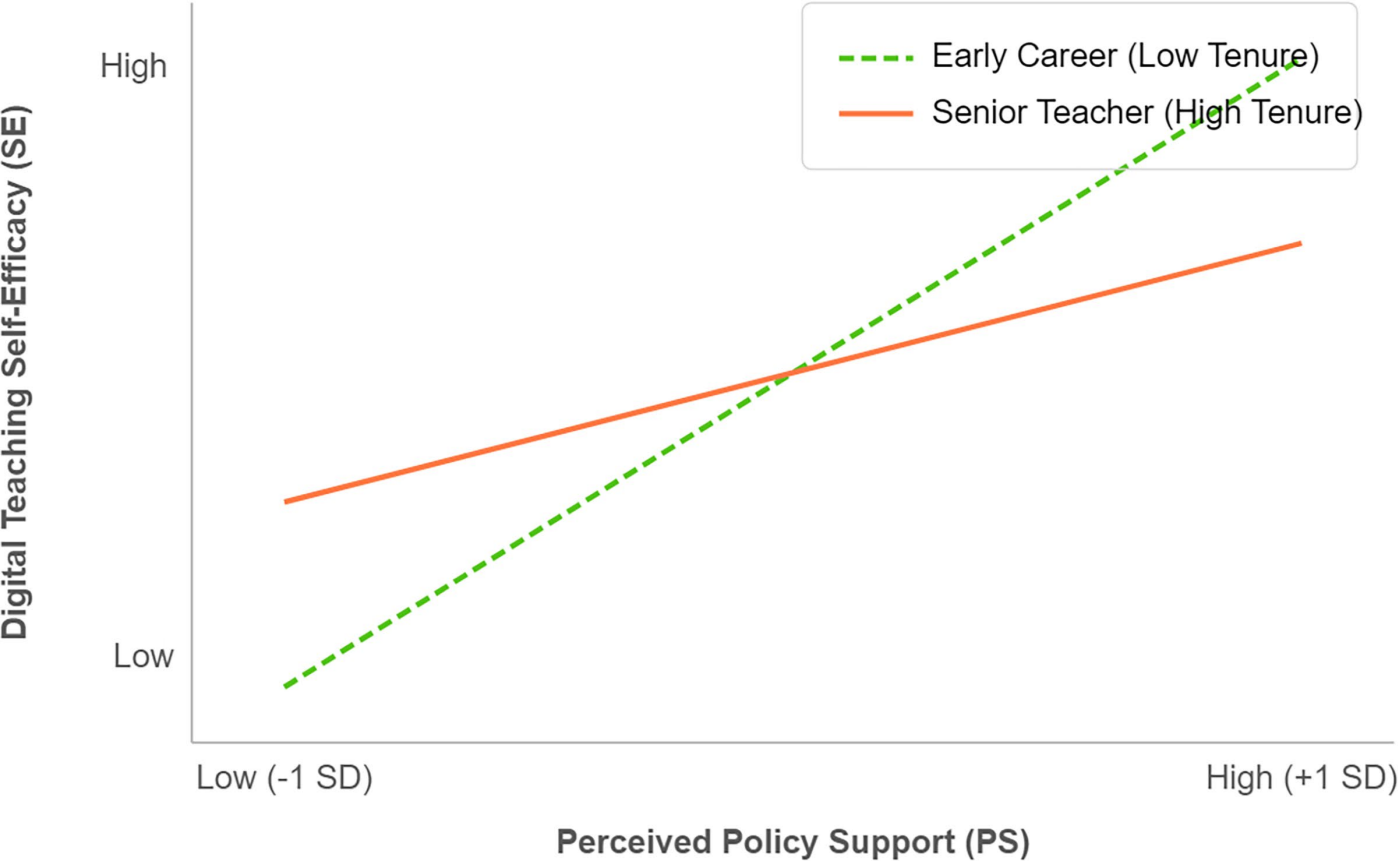
The moderating role of teaching experience in the relationship between policy support and self-efficacy. The figure illustrates the trend of perceived policy support’s influence on self-efficacy in digital teaching among two groups: senior teachers (with long teaching experience) and junior teachers (in early career).

To test whether teaching experience moderates the “policy support → self-efficacy” relationship, a hierarchical regression analysis was conducted. The results indicated that the interaction term was marginally significant (*β = −0.085, p = 0.062*). While not meeting the strict 0.05 threshold, as shown in Figure 4, this suggests a trend: policy support appears to have a stronger effect on enhancing the self-efficacy of early-career teachers compared to senior faculty.

## 5 Discussion

### 5.1 Theoretical contributions

This study makes several important theoretical contributions to the literature on educational digitalization, specifically revealing the distinctive drive mechanisms within the financial management context.

First, by integrating Organizational Support Theory and Social Cognitive Theory in the teacher path, we demonstrate that the impact of institutional policies is channeled critically through psychological empowerment. While the literature often discusses psychological empowerment and self-efficacy, our study clarifies their relationship in this context. We treat self-efficacy as a core, measurable component of psychological empowerment, specifically the competence dimension. Our findings suggest that it is this belief in one’s competence that acts as the primary mediator. Distinct from general humanities, the digital transformation of financial management requires mastering high-threshold tools (e.g., Python, Intelligent Financial Platforms). Our findings reveal that institutional support only influences pedagogical innovation when faculty digital self-efficacy—a key dimension of psychological empowerment—increases; without confidence in the competence to master industry-standard tools, structural interventions do not translate into behavioral change. This extends prior research by specifying the cognitive mechanism that links external support to behavioral outcomes in high-technicality disciplines.

Second, the student path analysis validates the Technology Acceptance Model while integrating it with deep learning theory, highlighting a “Value Rationality Logic.” Instead of merely stating that students engage when they see benefits, our findings, supported by stronger theoretical framing, suggest a more precise mechanism: Students demonstrate a deep approach when the perceived professional utility—for example, increased audit working paper accuracy, reconciliation automation, or CPA readiness—proves relevant to career goals. This mediating role of Perceived Usefulness challenges techno-determinist assumptions, indicating that students must perceive the genuine professional utility of these tools to trigger deep cognitive engagement.

Third, the dual-path framework offers a systemic perspective. By modeling teacher and student processes simultaneously, this study shifts the focus from “supply-side” resource allocation to “demand-side” psychological activation. The parallel mediating structures—psychological empowerment for teachers and value-rational evaluation for students—suggest that effective digital transformation requires addressing these distinct mindset barriers simultaneously. Furthermore, our heterogeneity analysis adds nuance, showing that policy effects vary by institutional tier and faculty career stage, underscoring that digitalization mechanisms are contingent on situational factors. This provides an empirical basis for moving beyond one-size-fits-all policies, a direction that resonates with recent systematic reviews calling for more comprehensive frameworks that link digital transformation initiatives to institutional-level outcomes and performance in higher education (Ramadania et al., 2024). The discussion is now strengthened with reference to more recent literature (Dulfer et al., 2025; Zheng and Shay, 2025), showing the contemporary relevance of our findings.

### 5.2 Practical implications

Based on the findings, this study offers targeted practical strategies for different stakeholders. For university administrators, the key is to shift from resource provision to psychological empowerment. This means not only investing in hardware but also designing teacher training programs that build confidence. For instance, creating “Intelligent Finance Faculty Workshops” that offer hands-on practice with industry tools like Python and RPA script libraries can directly boost self-efficacy, especially for early-career faculty. For educators, the implication is to make the value of digital tools explicit to students. Instead of just using technology, instructors should frame its use around career-oriented outcomes, such as building a portfolio of data analysis projects or preparing for professional certifications. This directly enhances perceived usefulness and motivates deep learning.

To ensure the external validity of these strategies, universities could implement them in other applied disciplines with similar tool-dependencies, such as accounting, business analytics, or engineering. Validating the model across different education systems (e.g., comparing outcomes in Chinese universities with those in Western institutions) would also be a valuable step to test the universality of the psychological mechanisms identified. The long-term impact of these interventions could be monitored through a structured evaluation plan with defined time horizons. For example, short-term metrics like classroom tool adoption rates and the quality of data-driven student assignments can be assessed on a semester-by-semester basis, while long-term outcomes, ultimately student internship and job placement rates in data-intensive roles, should be tracked annually.

Institutional Differentiation: The observed institutional-tier differences highlight a digital divide. Policymakers should allocate targeted support to under-resourced institutions (private and vocational colleges) to prevent the widening of inequality, potentially through cross-institutional “Virtual Teaching and Research Office” (teaching and research offices) collaborations.

### 5.3 Limitations and future research directions

This study has several limitations to consider.

First, the cross-sectional design precludes causal inference. Although the mediation models are theoretically grounded, longitudinal studies tracking teachers and students over time would strengthen claims regarding skill development and behavioral reinforcement.

Second, regarding data structure, this study used independent sampling and could not perform matched-pair analysis. This is a significant limitation as it prevents the analysis of direct interpersonal interaction effects (e.g., how a specific teacher’s high efficacy influences their own students’ perceptions). Future research should, if feasible, collect nested teacher-student paired data and employ Hierarchical Linear Modeling (HLM) to explore how teacher-level factors cross-levelly influence student-level outcomes, providing a more accurate depiction of the teaching and learning linkage mechanism.

Third, the sample, while diverse, has limitations in representativeness for certain subgroups. For instance, the number of faculty from “Double First-Class” universities (*N* = 38) and graduate students (*N* = 14) was relatively small. This limits the statistical power for heterogeneity comparisons involving these specific groups. Consequently, any conclusions drawn about these subgroups should be interpreted with caution as indicative trends rather than definitive findings. Future research should aim for a more balanced stratified sampling strategy to enhance the generalizability of subgroup analyses.

Finally, the study’s focus on financial management programs in China may limit generalizability. As suggested in our practical implications, future research should replicate the dual-path model in different academic fields (e.g., accounting, finance) and international settings to assess its robustness and cultural contingencies (Dulfer et al., 2025).

## 6 Conclusion

By constructing and testing a teacher-student dual-path model, this study aimed to systematically uncover the mechanisms through which digitalization policies affect teaching behaviors in university financial management programs. The conclusion is direct and clear: the success of digital transformation hinges on activating the right psychological drivers for both teachers and students.

For teachers, the path to innovation is paved with confidence. Our findings unequivocally show that institutional policy support is ineffective unless it successfully enhances teachers’ digital teaching self-efficacy. This highlights a “Confidence-Driven” mechanism, where psychological empowerment is the non-negotiable intermediate step.

For students, the motivation for engagement is rooted in value. A technologically rich environment is not enough. Students only commit to deep learning when they recognize the tangible utility of digital tools for their academic and professional goals. This “Value-Driven” mechanism underscores the importance of demonstrating relevance.

In summary, this study’s primary contribution is its shift in perspective from a technology-centric view to a human-centered one. It provides a robust theoretical model and empirical evidence demonstrating that effective digital transformation is fundamentally a psychological and cognitive endeavor. Policy recommendations should therefore focus less on mandating technology use and more on fostering teacher confidence and demonstrating value to students. By addressing these core human factors, institutions can translate their digital investments into meaningful pedagogical change. While acknowledging the study’s limitations, such as its cross-sectional design and non-matched samples, the findings offer a crucial roadmap for future policy and practice in educational digitalization.

## Data Availability

The original contributions presented in the study are included in the article/Supplementary material, further inquiries can be directed to the corresponding authors.
