# Supplementary material for: The impact of digitalization policies on teaching behaviors in university financial management programs: a teacher-student dual-path model
Source: Front Psychol. 2026 Apr 13;17:1773192. doi: 10.3389/fpsyg.2026.1773192 (PMC13111290; doi:10.3389/fpsyg.2026.1773192)
Supplement: Supplementary file 1 [file Data_Sheet_1.ZIP › Supplementary_Material_Survey_Instrument.docx]

### **Supplementary Material**

**Manuscript Title:** The Impact of Digitalization Policies on Teaching Behaviors in University Financial Management Programs: A Teacher-Student Dual-Path Model

**Document Description:** This supplementary file contains the complete survey instruments used for data collection in the aforementioned manuscript. The questions were originally presented to participants in Chinese. For the purpose of this review, they have been translated into English. The file is divided into two parts: Part A (Teacher Questionnaire) and Part B (Student Questionnaire).

### **Part A: Teacher Questionnaire**

**1. Informed Consent**

*The following text was presented to each participant at the beginning of the survey (translated from Chinese).*

**Dear Faculty Members,**

Greetings!

We are a research team from [Blinded for Review]. We cordially invite you to participate in an academic study titled “ The Impact of Digitalization Policies on Teaching Behaviors in University Financial Management Programs.”

Research Purpose: This study aims to deeply understand the current status of teaching in Financial Management programs and the authentic experiences of faculty and students against the backdrop of educational digitalization. The findings will provide a scientific reference for optimizing teaching policies and practices.

Participation Details:

Voluntary Participation: Your participation in this survey is entirely voluntary. You have the right to interrupt or withdraw at any time without any negative consequences.

Confidentiality & Anonymity: The survey is collected anonymously. The system will not record your name, IP address, or any personally identifiable information. All data will be used solely for aggregate statistical analysis, and your individual responses will be strictly confidential.

Potential Risks: This study involves no sensitive personal privacy issues and is not expected to cause any risk or discomfort.

Estimated Time: Completing the questionnaire will take approximately 5-7 minutes.

If you have read and understood the above information and agree to participate in this study, please click “Next” to begin.

We sincerely thank you for your trust and valuable time!

**2. Screening Questions**

SQ1. What is your current role?

A. University Faculty
B. Other

SQ2. Do you currently (or have you within the last three years) teach courses in Financial Management (or related programs such as Accounting or Auditing)?

A. Yes
B. No

**3. Measurement Scales**

*All items below were measured on a 7-point Likert scale (1 = Strongly Disagree, 7 = Strongly Agree).*

**3.1. Perceived Policy Support (PS)**

Operational Definition: The degree to which teachers perceive that their institution (university or school) provides resources, incentives, and a supportive environment for them to conduct digital teaching.
Source: Adapted from Eisenberger et al. (1986).

Items:

(PS1) I feel that my school/college’s policies clearly encourage us to explore digital teaching methods.

(PS2) The school provides sufficient training related to digital teaching skills.

(PS3) I can conveniently access technical support services from the school when needed.

(PS4) I believe the school’s evaluation and incentive mechanisms adequately reflect my efforts and contribution to digital teaching.

**3.2. Digital Teaching Self-Efficacy (SE)**

Operational Definition: Teachers’ belief in their capability to successfully organize and execute courses of action required to accomplish specific digital teaching tasks within the financial management program context.
Source: Adapted from Bandura (1997).

Items:

(SE1) I am confident in my ability to proficiently operate the various digital platforms and software required for teaching this major.

(SE2) I believe I can utilize digital tools to design high-quality financial management courses.

(SE3) I can remain calm and resolve sudden technical issues encountered during teaching.

(SE4) I have the ability to effectively integrate cutting-edge financial technologies (e.g., RPA, Big Data analytics) into my teaching.

(SE5) I believe I can effectively stimulate student interest and engagement through digital means.

**3.3. Innovative Teaching Behavior (IB)**

Operational Definition: The intentional generation, promotion, and application of new, digital-related ideas, methods, and processes within the teaching role.
Source: Adapted from Janssen (2000).

Items:

(IB1) I actively seek out and learn about new digital teaching tools or methods applicable to this major.

(IB2) I frequently contemplate how to better integrate existing teaching content with digital methods.

(IB3) I am willing to share insights and experiences regarding digital teaching with colleagues within my team (e.g., teaching and research office).

(IB4) I devote personal time to designing or refining digital teaching resources related to Financial Management (e.g., online cases, micro-lectures).

**3.4. Attention Check Item**

Item: To ensure the validity of the questionnaire, please select “6” for this item.

**4. Demographic Information**

Gender: Male / Female

Age: [Open-ended]

Highest Degree: Bachelor’s / Master’s / Doctoral

Professional Title: Assistant / Lecturer / Associate Professor / Full Professor

Teaching Experience: [Years]

Institution Tier: “Double First-Class” Univ. / Public Undergraduate / Private or Independent College / Higher Vocational College

### **Part B: Student Questionnaire**

**1. Informed Consent**

*The following text was presented to each participant at the beginning of the survey (translated from Chinese).*

**Dear Students,**

Greetings!

We are a research team from [Blinded for Review]. We cordially invite you to participate in an academic study titled “The Impact of Digitalization Policies on Teaching Behaviors in University Financial Management Programs.”

Research Purpose: This study aims to deeply understand the current status of teaching in Financial Management programs and the authentic experiences of faculty and students against the backdrop of educational digitalization. The findings will provide a scientific reference for optimizing teaching policies and practices.

Participation Details:

Voluntary Participation: Your participation is entirely voluntary. You may withdraw at any time.

Confidentiality & Anonymity: Responses are anonymous. No personally identifiable information is recorded. Data is used for statistical analysis only.

Potential Risks: No known risks; the study involves no sensitive privacy issues.

Estimated Time: Approximately 5-7 minutes.

If you agree to participate, please click “Next”.

Thank you for your time!

**2. Screening Questions**

SQ1. What is your current status?

A. University Student (Undergraduate/Graduate)
B. Other

SQ2. Is your major Financial Management (or a related field such as Accounting or Auditing)?

A. Yes
B. No

**3. Measurement Scales**

*All items below were measured on a 7-point Likert scale (1 = Strongly Disagree, 7 = Strongly Agree).*

**3.1. Perceived Digital Learning Environment (DLE)**

Operational Definition: A student’s overall perception of the quality and richness of the policy-driven digital learning platforms, resources, interactions, and support.
Source: Developed for this study.

Items:

(DLE1) I find that the school’s online learning platforms (e.g., Chaoxing, Zhihuishu) are stable and user-friendly.

(DLE2) In my major studies, instructors provide rich digital learning resources (e.g., videos, case databases, electronic materials).

(DLE3) I feel that instructors skillfully use digital tools for class interaction (e.g., voting, discussions).

(DLE4) When I have major-related questions, I can receive timely answers and feedback from teachers via digital channels.

(DLE5) The school ensures smooth access to professional financial software and databases.

**3.2. Perceived Usefulness (PU)**

Operational Definition: The degree to which a student believes that using a particular digital system or tool would enhance their learning performance and professional skills.
Source: Adapted from Davis (1989).

Items:

(PU1) I believe that using digital tools and resources significantly improves my learning efficiency in Financial Management.

(PU2) Digital learning methods help me better understand abstract and complex concepts in Financial Management.

(PU3) I believe mastering these digital learning skills is beneficial for my future job seeking and career development.

(PU4) Overall, I believe digital learning methods are very useful for enhancing my professional competence.

**3.3. Deep Learning Behavior (DL)**

Operational Definition: A student’s tendency to engage in higher-order cognitive activities, such as critical thinking and knowledge integration.
Source: Adapted from Biggs et al. (2001).

Items:

(DL1) When studying Financial Management courses, I often try to understand the underlying principles rather than just memorizing conclusions.

(DL2) I actively connect knowledge learned in different courses to form a complete knowledge system.

(DL3) I think critically about the viewpoints in the digital resources provided by teachers and consult more materials for verification.

(DL4) I enjoy exploring a specific topic of interest deeply in the course, even if it is not a mandatory requirement by the teacher.

**3.4. Attention Check Item**

Item: To ensure the validity of the questionnaire, please select “2” for this item.

**4. Demographic Information**

Gender: Male / Female

Academic Year: Freshman / Sophomore / Junior / Senior / Graduate Student

Institution Tier: “Double First-Class” Univ. / Public Undergraduate / Private or Independent College / Higher Vocational College

Daily Digital Learning Time: [Hours]

*End of Supplementary Material*
